# The need for a broad perspective when assessing value-for-money for out-of-hours primary care

**DOI:** 10.1017/S1463423624000318

**Published:** 2024-09-20

**Authors:** Jane Phiri, Stefan Morreel, Diana De Graeve, Hilde Philips, Philippe Beutels, Veronique Verhoeven, Lander Willem

**Affiliations:** 1 Department of Family Medicine and Population Health (FAMPOP), University of Antwerp, Antwerp, Belgium; 2 Department of Economics, University of Antwerp, Antwerp, Belgium; 3 Centre for Health Economic Research and Modelling Infectious Diseases (CHERMID), University of Antwerp, Antwerp, Belgium

**Keywords:** access (to care), continuity of care, economic evaluation methods, out-of-hours care, outcome measures, primary care utilisation, primary healthcare, productivity, societal perspective, welfare

## Abstract

**Background:**

Out-of-hours primary care (OOH-PC) has emerged as a promising solution to improve efficiency, accessibility, and quality of care and to reduce the strain on emergency departments. As this modality gains traction in diverse healthcare settings, it is increasingly important to fully assess its societal value-for-money and conduct thorough process evaluations. However, current economic evaluations mostly emphasise direct- and short-term effect measures, thus lacking a broader societal perspective.

**Aim:**

This study offers a comprehensive overview of current effect measures in OOH-PC evaluations and proposes additional measures from the evaluation of integrated care programmes.

**Approach and Development:**

First, we systematically identified the effect measures from published cost-effectiveness studies and classified them as process, outcome, and resource use measures. Second, we elaborate on the incorporation of ‘productivity gains’, ‘health promotion and early intervention’, and ‘continuity of care’ as additional effects into economic evaluations of OOH-PC. Seeking care affects personal and employee time, potentially resulting in decreased productivity. Challenges in taking time off work and limited access to convenient care are often cited as barriers to accessing primary care. As such, OOH-PC can potentially reduce opportunity costs for patients. Furthermore, improving access to healthcare is important in determining whether people receive promotional and preventive services. Health promotion involves empowering people to take control of their health and its determinants. Given the unscheduled nature and the fragmented or rotational care in OOH-PC, the degree to which interventions and modalities provide continuity should be monitored, assessed, and included in economic evaluations. Continuity of care in primary care improves patient satisfaction, promotes adherence to medical advice, reduces reliance on hospitals, and reduces mortality.

**Conclusion:**

Although it is essential to also address local settings and needs, the integration of broader scope measures into OOH-PC economic evaluations improves the comprehensive evaluation that aligns with welfare gains.

## Background and aims

Global commitment to out-of-hours primary care (OOH-PC) and recognition of the importance of this healthcare format is increasing (Hong *et al*., 2020; Steeman *et al*., 2020). Additionally, research has demonstrated that its implementation has the potential to improve the quality of care, optimise efficiency, and reduce the strain on emergency departments (EDs) (Mohsin *et al*., 2007; Guttmann *et al*., 2011; Whittaker *et al*., 2016; Hong *et al*., 2020; Allen *et al*., 2021). Although there is no consensus on the definition of ‘appropriate’ or ‘inappropriate’ use of the ED, several studies find that many medical problems presented in the ED could be managed in a primary care setting, as they do not always require specialist care (Derlet & Ledesma, 1999; Carret *et al*., 2007; Durand *et al*., 2011; Kraaijvanger *et al*., 2016).

OOH-PC is operationally defined in terms of time frame, as primary care delivered on weekdays outside business hours, approximately from 6:00 PM to 8:00 AM, during weekends or public holidays (O’Donnell *et al*., 2015). Such care is classified as unscheduled, meaning that no appointment or forward planning is arranged beforehand (O’Donnell *et al*., 2015). However, there is a discussion in the literature on whether out-of-hours care should only provide urgent care or include non-urgent care as well (Keizer *et al*., 2015; O’Donnell *et al*., 2015; Barnes *et al*., 2022). Nonetheless, for this paper, we adopted a broad and commonly accepted definition of OOH-PC based solely on the time frame, specifically excluding care delivered in the ED or other secondary or tertiary levels.

OOH-PC encompasses several models of delivery, such as practice-based services in which physicians within an individual or group practice look after their own and each other’s patients during OOH times (Berchet & Nader, 2016). Another model is general practice cooperatives (GPCs), which are large-scale self-organised groups of general practitioners (GPs) providing out-of-hours care in a region (Berchet & Nader, 2016; Colliers *et al*., 2017). Additionally, there are also retail or medical clinics located within grocery stores or pharmacies, typically staffed by nurses or other health professionals (Berchet & Nader, 2016).

Many countries have now adopted OOH-PC (Steeman *et al*., 2020), necessitating its inclusion in the decision-making process regarding future investments. For decisions that aim to maximise welfare, the perspectives of the healthcare payer, the hospital, and society are informative, in which the latter incorporates the full range of relevant costs and effects, including patient long-term outcomes and productivity losses (Byford & Raftery, 1998). However, evidence on the economic evaluation of OOH-PC service delivery is scarce and limited, despite numerous suggestions to robustly assess the causal impact of improving access to primary care on the use of other services, outcomes, and costs (WHO & UNICEF, 2022). Some studies in this domain have focused only on the estimation of cost implications (Brogan *et al*., 1998; Scott *et al*., 2004; O’Dowd, 2006; van Uden *et al*., 2006; Eichler *et al*., 2010; Moth *et al*., 2013; Lin *et al*., 2021; Morreel *et al*., 2022). Others have incorporated effects by including immediate health system effects that manifest within a short time frame of patient presentation, such as admissions or practice attendance (Hansen & Munck, 1998; Lattimer *et al*., 2000; Moore *et al*., 2021; Flaherty *et al*., 2022). Although OOH-PC is commonly considered a short-term intervention, it is also crucial to capture long-term societal outcomes (Deidda *et al*., 2019).

This study aims to first provide a comprehensive overview of the effect measures currently used in the economic evaluations of OOH-PC interventions and, second, propose an additional set of societal effect measures to capture the broader economic value of this healthcare service. The additional measures relevant to OOH-PC are identified and discussed based on an overview of economic evaluations of integrated care programmes.

## Approach and development

### Systematic literature search

We consulted PubMed, SCOPUS, Web of Science, EconLit, Cochrane reviews, NHSEED, and Health Technology Assessment databases for articles that performed economic evaluations of OOH-PC. See Appendix A for details on the search strategy. Eligibility criteria were agreed upon by two researchers (JP and LW) using the PICOTS framework (Population, Intervention, Comparator/Context, Outcome, Timing, and Study Design/Setting) to define inclusion and exclusion criteria (Table 1).

**Table 1.** PICCOTS

PICCOTS	Included	Excluded
Participants	Any	–
Intervention	Out-of-hours consultations/services as the component of analysis (this includes OOH telephone consultations, telephone triage, health facility visits, and home visits) There is no restriction on out-of-hours mode of delivery and/or organisation of care	Services/interventions where out-of-hours is not the primary component of analysis, but rather the study setting. For example, a study comparing alternative rehabilitation services provided out-of-hours
Comparator	Any	–
Context	Articles of any design reporting original research including empirical findings and protocols	Books, chapters, letters, comments, or editorials
Outcome	Any (natural/physical units, utility-score-based measures, monetary units, etc)	–
Timing	No time restriction	–
Study design	Full economic evaluations, including both costs and effects (cost-effectiveness analyses (CEA), cost–utility analyses, cost–benefit analyses, cost-consequence analyses (CCA), and return on investment)	Partial economic evaluations: including costs only (cost analysis)
Setting	Primary care settings (excluding emergency departments)	Secondary and tertiary care setting. This includes hospitals, emergency departments, urgent care facilities, and other specialised care facilities

The online search identified 2717 unique results; we identified one additional article by screening the references of seven systematic reviews on OOH-PC effectiveness. After removing 299 duplicates, we selected 101 from the title and abstract screening, and finally, we included 13 in this overview after reading the full text. See Appendix B for the PRISMA diagram.

We evaluated the quality of the 13 studies using the Consensus Health Economic Criteria (CHEC) list, which is a 19-point checklist researchers use to evaluate the reporting and methodology of published economic evaluations (Evers *et al*., 2005). Of the studies, 10 received a score greater than or equal to 75%, while three received a score of between 51% and 74%. (See Appendix C for the evaluation approach and the assessment results). Note that scores cover both the quality of the study conducted and the completeness of reporting. However, if a paper does not discuss context because it seemed obvious, we flagged the corresponding item as not applicable and excluded the item from the denominator following the CHEC guidelines.

The 13 included studies comprised 11 research papers that evaluated the cost-effectiveness and efficiency of various OOH-PC healthcare interventions and modalities (Broekman *et al*., 2017; Chesteen *et al*., 1986; Flaherty *et al*., 2022; Flynn, 1998; Hansen & Munck, 1998; Lattimer *et al*., 2000; Moe *et al*., 2019; Moore *et al*., 2021; Patwardhan *et al*., 2012; Poole *et al*., 1993; Sterner *et al*., 2012) and two protocol papers proposing full economic evaluations (Reuter *et al*., 2016; Wijers *et al*., 2012). Since our focus was on the effect measures and not on the results, these protocols are sufficient and studies that published the results were not included.

Among these studies, five compared alternative ways of providing OOH-PC (Broekman *et al*., 2017; Hansen & Munck, 1998; Lattimer *et al*., 2000; Reuter *et al*., 2016; Wijers *et al*., 2012), while seven compared OOH-PC with ED or with a ‘no OOH-PC’ scenario (Chesteen *et al*., 1986; Flaherty *et al*., 2022; Flynn, 1998; Moe *et al*., 2019; Moore *et al*., 2021; Poole *et al*., 1993; Sterner *et al*., 2012). Additionally, one study compared OOH-PC with urgent care centres, primary care physicians, ED, and without intervention (Patwardhan *et al*., 2012). In total, these studies evaluated six types of OOH-PC: telephone support, nurse-delivered care, telemedicine, home delivery, late night/weekend/holiday clinics for alcohol intoxication, after-hours clinics/family practices or urgent care centres, and jointly operating ED and GP care. See Appendix D for a further description of the studies.

### Measures previously used in the economic evaluation of OOH-PC

With the formalisation of OOH-PC implementation, numerous published studies have concentrated on its effectiveness. In the upper section of Table 2, we have compiled common effectiveness measures based on the findings of seven systematic reviews (Foster *et al*., 2020; Fry, 2011; Garratt *et al*., 2007; Hong *et al*., 2020; Huibers *et al*., 2011; Leibowitz *et al*., 2003; O’Donnell *et al*., 2015). In contrast, the literature on cost-effectiveness is comparatively limited and systematic reviews are absent (O’Donnell *et al*., 2015). Therefore, we performed a systematic search of the literature and retrieved 13 studies as outlined in Section 2.1. The lower section of Table 2 compiles the effect measures based on the findings of these studies.

**Table 2.** Overview of effect measures related to the process, patient outcome, and healthcare resource use in the effectiveness and economic evaluation of OOH-PC interventions

Process measures	Patient outcome measures	Health resource measures
**Effect measures used in the effectiveness evaluation of OOH-PC interventions**		
• Access to healthcare services • Quality/appropriateness of advice given • Safety • Waiting time to consultation/treatment • Provider satisfaction and morale • Stakeholder perceptions (GPCs, ED, and national policymakers)	• Mortality • Patient experience • Health status	• Utilisation of the Emergency department • Number of patients seen • Consultation time • Number of consultations managed through medical advice, telephone calls, surgery, home visits, face-to-face consultation with a GP or other healthcare professional at a primary care centre. • Utilisation of other healthcare services • Utilisation of ambulance services • Referral to the emergency department • Hospital admissions • Follow-up/subsequent visits • Management without a GP or referral • Drug prescriptions
**Effect measures used in the economic evaluation of OOH-PC interventions**		
• Quality and safety of care • Knowledge and competencies of providers • Ambulance response within a prescribed time frame • Feasibility of substituting cadres of staff delivering care, for example, transitioning from general practitioners to nursing practitioners • Provider workload	• Patient satisfaction • Mortality • Health status (EQ-5D) • Health-related quality of life (HRQoL) (disability-adjusted life years)	• Length of stay (timespan between registration and discharge outpatients) • ED visits (initial and follow-up) • Type of consultation • Emergency hospital admissions • Hospital admissions • Hospital admission length of stay • Number of patients seen by a clinician (workload indicator) • Attendance at a primary care centre/practice • GP home visits • Drug prescriptions • Number of tests and investigations • Number of referrals to other facilities

Table 2 shows the effect measures related to process, patient outcomes, and healthcare resource used to evaluate OOH-PC interventions’ effectiveness and economic evaluations. According to the Donabedian paradigm, we categorise the measures into three groups according to the consensus-based entity submission types outlined in the Centers for Medicare & Medicaid Services Measures (CSM) Inventory Tool (Measures Management Systems, 2023). A process measure refers to the evaluation of specific steps, procedures, and environment essential to provide quality care (Measures Management Systems, 2023). When a process measure is managed effectively, the likelihood of achieving the desired outcome increases. For example, more general access to healthcare services can contribute to reduced mortality (Measures Management Systems, 2023). A patient outcome measure focuses on assessing a patient’s health status or any changes in their welfare resulting from healthcare interventions (Measures Management Systems, 2023). Examples of outcome measures include mortality rates and gains in quality-adjusted life years (QALYs). A measure of healthcare resource use quantifies the utilisation of healthcare services expressed in terms of natural units (Measures Management Systems, 2023). This encompasses various aspects, including diagnoses, procedures, or healthcare encounters, and can be exemplified by metrics such as the number of GP visits (Measures Management Systems, 2023).

As shown in the upper part of Table 2, effectiveness studies mostly include the use of healthcare resources, a few patient outcome measures, and a limited number of process measures. Similarly, the lower part of Table 2 shows that OOH-PC economic evaluations focus on the use of healthcare resources and a few patient outcome measures, while process measures remain relatively underutilised.

### The need for a broader scope for OOH-PC

OOH-PC falls within the umbrella of integrated care, and it is necessary to measure and evaluate its broader effects at various levels. Given this, OOH-PC has been shown to improve access to care for those in need, reduce ED visits, and promote efficiency (Dent, 2010; Lowe *et al*., 2005; Piehl *et al*., 2000). Moreover, integrated care represents care that is coordinated across professionals, facilities, and support systems, is continuous over time, and is responsive to people’s needs, values, and preferences (Schneider *et al*., 2011). It encompasses treatment plans, methods, and models of care that enable improvement in patient experience, promote efficient service delivery, reduce healthcare expenditures, and improve population health through enhanced coordination and continuity of care (Plochg *et al*., 2009; Shaw *et al*., 2011). These types of interventions impact various outcomes at various levels, necessitating the measurement and evaluation of multiple outcomes (Tsiachristas *et al*., 2016). Furthermore, they alter existing care processes and pathways and impact providers, patients, and communities (Baxter *et al*., 2018), henceforth the recommendation for a broader evaluation. A broad scope of evaluation has been applied to various areas of integrated care for public decision-making (Nolte & Pitchforth, 2014).

Table 3 presents an overview of the effect measures used previously or recommended for use to measure the effects of integrated care in economic evaluations. We derived this information from two systematic reviews and several individual research studies (KPMG, 2018; Nolte & Pitchforth, 2014; Rocks *et al*., 2020; Steuten *et al*., 2006; Tsiachristas *et al*., 2013). Like Table 2, the measures are categorised into process, healthcare resources, and patient outcome. Although healthcare resource use measures are important indicators of health system performance, it is also essential to consider patient-centred outcomes, including population health, patient, or community well-being. The combination of patient-centred and process-related measures allows for a more comprehensive evaluation.

**Table 3.** Overview of effect measures related to the process, patient outcome, and healthcare resource use in the economic evaluation of integrated care interventions

Process measures	Patient outcome measures	Health resource measures
• Continuity of care − Access to healthcare services − Continuous relationship between patient and provider − Referral behaviour − Patients receiving routine follow-up − Effective communication and coordination between healthcare providers − Effective feedback loops − Use of monitoring systems • Adherence to guidelines • Efficient flow from consultation to admission • Health promotion − Early intervention	• Mortality • Clinical health status • Diagnosis and management of disease • Functional health status • Health-related quality of life • Satisfaction with healthcare • Frequency of complications and exacerbations • Self-efficacy • Patient time and travel • Productivity loss	• Informal caregivers time • Hospital admissions • Hospital length of stay • ED consultations • Regular outpatient visits • Drug prescription

### Additional key measures relevant for OOH-PC economic evaluations

Comparison of Tables 2 and 3 reveals that integrated care interventions employ effect measures beyond immediate outcomes. These effects are more extensive, covering broader health system and patient effects compared to those used in OOH-PC. Consequently, we advocate for the inclusion of additional effects in the evaluation of OOH-PC to improve the comprehensive understanding of the economic value. Additionally, it will create synergy between OOH-PC and conceptually similar integrated care interventions, which can complement and/or substitute OOH-PC.

Following this mapping of the effects of integrated care initiatives and OOH-PC, we identified eight additional effect measures for integrated care initiatives not yet used in OOH-PC. These measures include two process measures, one healthcare resource use measure, and five patient measures. Specifically, two identified process measures are ‘continuity of care’ and ‘health promotion’, while the additional measure of using healthcare resources is ‘informal caregiver time’. The innovative patient outcome measures for OOH-PC studies are ‘diagnosis and disease management’, ‘frequency of complications and exacerbations’, ‘self-efficacy’, ‘patient travel and time’, and ‘productivity loss’. While all eight measures are relevant for the economic evaluation of OOH-PC, in this study, we narrow our discussion to three key measures where the out-of-hours aspect is crucial and distinguishes itself from within-hours care: ‘continuity of care’, ‘health promotion’, and ‘productivity loss’. The added value of each selected measure is discussed in the following paragraphs. Among the five outcome measures not highlighted here, such as ‘diagnosis and disease management’, ‘patient travel and time’, or ‘informal caregivers time’, we believe that the difference with regular primary care is limited. However, all identified outcome measures that are currently absent from published economic evaluations of OOH-PC offer valuable insights into clinical outcomes or well-being, while depicting costs for the healthcare payer, the patient, or society.

Table 4 shows a comprehensive summary of the effect measures for OOH-PC evaluations. It lists additional measures proposed from integrated care and those used in previous OOH-PC evaluations. The effect measures that we emphasise in the main text are coloured red. In the subsequent paragraphs, we elucidate and delve into the proposed supplementary effect measures, offering illustrative examples from related domains.

**Table 4.** Comprehensive summary of effect measures related to process, patient outcomes, and healthcare resource use for OOH-PC evaluation

Process measures	Patient outcome measures	Health resource measures
• Access to healthcare services • Quality/appropriateness of advice given • Safety • Provider satisfaction and morale • Stakeholder perceptions (GPCs, ED, and national policymakers) • Quality and safety of care • Knowledge and competencies of providers • Ambulance response within a prescribed time frame • Feasibility of substituting cadres of staff delivering care, for example, transitioning from general practitioners to nursing practitioners • Provider workload • Continuity of care − Continuous relationship between patient and provider − Referral behaviour − Patients receiving routine follow-up − Effective communication and coordination between healthcare providers − Effective feedback loops − Use of monitoring systems • Adherence to guidelines • Efficient flow from consultation to admission • Waiting time to consultation/treatment • Health promotion − Early intervention	• Mortality • Patient experience/satisfaction • Waiting time for treatment • Health status (EQ-D5) • HRQoL (DALY/QALY) • Clinical health status • Diagnosis and management of disease • Functional health status • Frequency of complications and exacerbations • Self-efficacy • Patient time and travel • Productivity loss	• Utilisation of the emergency department • Number of patients seen • Consultation time • Number of consultations managed through medical advice, telephone calls, surgery, home visits, and face-to-face consultation with a GP or other healthcare professional at a primary care centre. • Utilisation of other healthcare services • Utilisation of ambulance services • Referral to the emergency department • Hospital admissions • Follow-up/subsequent visits • Management without a GP or referral • Drug prescriptions • Length of stay (timespan between registration and discharge) • Type of consultation • Emergency hospital admissions • Hospital admission length of stay • Attendance at a practice • GP home visits • Drug prescriptions • Number of tests and investigations • Number of referrals to other facilities • Informal caregiver’s time • Regular outpatient visits

#### Productivity losses averted (opportunity cost of seeking healthcare)

Economic evaluations must consider the opportunity cost of seeking care, which can affect personal and workforce time, resulting in productivity loss (Ray *et al*., 2015; Weinstein *et al*., 1996). From the patient’s perspective, productivity loss contributes to wage loss and causes undesirable experiences when seeking care (Handley & Hollander, 1999; NHS Primary Care Commissioning, 2012). Additionally, the difficulty of taking time off work and the lack of access to convenient care are two factors often cited as barriers to accessing regular-hour primary healthcare (Friedberg *et al*., 2010; NHS Primary Care Commissioning, 2012). In contrast, these two factors are facilitators of increased use of OOH-PC (Zhou *et al*., 2015). Therefore, OOH-PC offers patients a convenient avenue to access primary care outside of working hours, helping to avoid wage and time losses. This is also relevant for caregivers responsible for caring for the disabled, elderly, and young children. OOH-PC grants these individuals the ability to refrain from taking time away from work and sacrificing their caregiving obligations concerning their care recipients’ healthcare needs. This also applies to students and school-aged children. Research indicates that students who rely on public clinics often miss entire days of school per appointment (Kornguth, 1990). Furthermore, research findings indicate that children frequently use OOH-PC in European settings (Huibers *et al*., 2011), and its use may help reduce school absenteeism (Institute of Medicine, 1997). Productivity gains in this group can be achieved by minimising absenteeism among students and school-aged children. Therefore, it is important to include the productivity loss avoided in the evaluation of OOH-PC, as it represents an important positive gain for patients, caregivers, students, and school-aged children. This gain is an important influence in this domain, and it is crucial not to underestimate this potential advantage. Additionally, it is important to consider the productivity loss caused by using OOH-PC. Furthermore, opportunity costs are important due to the increased recognition of patient-centred care (Baker, 2001). Subsequently, this has led to an emphasis on innovative healthcare delivery options that reduce the time burden (Ray *et al*., 2015).

Productivity gains have been included in economic evaluations of telemedicine (Patel *et al*., 2023; Snoswell *et al*., 2020), which is a conceptually similar intervention to OOH-PC. Although not delivered specifically outside of working hours, telemedicine also provides convenient, patient-centred care that improves access to care and reduces time burden. Additionally, it seeks to reduce the inefficient use of higher-level facilities, including EDs (Sun *et al*., 2020). By providing the necessary services by electronic means, telemedicine serves individuals who have difficulties in making appointments or may not have the time, resources, or motivation to travel to traditional clinics (Zhang *et al*., 2021). Eliminating these logistical difficulties results in decreased patient productivity loss in the economic evaluation of telemedicine (Agha *et al*., 2002; Kubes *et al*., 2021). Estimation of productivity losses or gains in economic evaluations is complex and uses various measures. These include the human capital approach, friction cost approach, or multiplier approach, which can account for productivity in natural units, opportunity costs, and replacement costs (Jiang *et al*., 2022). Because there is no universally accepted measure, the choice of approach must depend on existing national guidelines while considering available information in a specific context.

#### Access to health promotion services and early intervention

Health prevention involves taking action to avoid the onset of disease and associated risk factors (Radhakrishnan, 2017). These actions include vaccinations, prophylaxis, education of people about behavioural and medical health risks, and disease detection (Radhakrishnan, 2017). Similarly, health promotion involves empowering people to take control of their health and its determinants (Radhakrishnan, 2017). Examples of these include dietary and nutritional interventions, interventions to mitigate social ills such as domestic violence, and interventions to promote sexual and reproductive health, such as family planning services (Radhakrishnan, 2017).

Reliable access to health-promotive and preventive services in primary healthcare is important for improving health outcomes and reducing the financial burden of treating diseases (Hostetter *et al*., 2020). Providing these services promotes timely diagnosis and treatment, increasing the chances of success (World Health Organization, 2018). An efficient and effective primary healthcare system must provide promotive and preventive services (Van Weel & Kidd, 2018). However, despite the benefits, many settings do not have optimal access to these recommended services (Borsky *et al*., 2018; Levine *et al*., 2019).

Having access to primary healthcare is a crucial factor that determines whether people receive promotive and preventive services (Xu, 2002; Friedberg *et al*., 2010). OOH-PC is efficient in improving access to primary care (Hong *et al*., 2020). Therefore, OOH-PC represents a suitable resource for those who would otherwise not seek care at a regular-hour health facility for the necessary services and check-ups. This is particularly so in settings or modalities where OOH-PC permits or is used for such services. Without such care and services, delayed diagnoses and treatment can lead to complicated disease management (World Health Organization, 2018). Given the profound role that OOH-PC can play in delivering promotive and preventive services, a robust economic assessment must consider incorporating these benefits. Their omission could produce a conservative estimate of the economic benefits of OOH-PC.

Improved access to preventive and promotive health services has been used in conceptually similar interventions. For example, a recent cost–benefit analysis (CBA) from Australia examined nurse-led primary healthcare facilities and explored how they impacted the provision of promotive and early interventions (KPMG, 2018). Similarly, economic evaluations of mobile and community clinics included the adoption of preventive and promotive services (Liu *et al*., n.d.; Oriol *et al*., 2009; Stillmank *et al*., 2019).

In the context of a societal evaluation, it has been proposed that the value of healthcare services should not be limited to their value to patients alone (Culyer *et al*., 2018). While the well-being of patients remains a focal point, it is imperative to incorporate the perspectives of healthcare providers to gain a comprehensive understanding of the practical implications of the delivery of healthcare services. This is of relevance for health promotion and preventive services, as providers often face heavy workloads (Smits *et al*., 2014; Royal College of General Practitioners, 2019). This can limit their capacity to offer non-urgent care on an unscheduled basis, as they must prioritise urgent care. Thus, in addition to the potential benefits to the patient, it is necessary to quantify the deterrent effects, if possible, and consider the viewpoint of service providers as relevant and appropriate measures of value regarding health-promoting and preventive services in the OOH-PC domain. However, striking a balance between the potentially opposing needs and perspectives of patients and providers is a complex but indispensable task.

Measuring the impact of an intervention on the acceptance of promotive or preventive services involves assessing whether the number of visits related to these services has changed since the implementation of that intervention (KPMG, 2018). However, establishing the long-term economic impact of preventive care and health promotion is challenging for several reasons. At the population level, it can be challenging to isolate the impact of individual interventions because the impacts are bundled or extend beyond healthcare. Additionally, some settings may encounter challenges with missing or inferior data, or practical/ethical/privacy problems when linking data from several sources or contacts (Colliers *et al*., 2016). In cases where data are accessible, the estimation of QALYs can be considered using clinically preventable burden scores, as has been demonstrated in previous research (Maciosek *et al*., 2017; Stillmank *et al*., 2019).

#### Continuity of care

Relationship continuity of care is the maintenance of continuous and sustained relationships between patients and healthcare professionals (Gulliford *et al*., 2006; Hill & Freeman, 2011). Research findings demonstrate that establishing a good, trust-based, and long-term relationship with a primary care physician of one’s choice can lead to improved health outcomes, better quality of care, and reduced healthcare expenses (Starfield *et al*., 2005). On the other hand, management continuity refers to care systems facilitated by integration, coordination, and the sharing of information between different providers (Gulliford *et al*., 2006; Hill & Freeman, 2011). Continuity of care in primary healthcare, both in relationship and in management, is beneficial for patients, clinicians, and health systems. It leads to increased patient satisfaction, improved care for chronic patients, increased use of preventive care, promoted adherence to medical advice, reduced dependency on hospitals, and reduced mortality (Gray *et al*., 2018; Sidaway-Lee *et al*., 2019).

Despite its benefits, continuity of care remains low in some primary care settings and is rarely measured (Sidaway-Lee *et al*., 2019). Measurement and comparison of continuity rates among providers can, in turn, improve continuity (Kontopantelis *et al*., 2013). Furthermore, ensuring patient-centredness and continuity of care are crucial attributes of a well-functioning primary care system. Therefore, it is important to establish whether an intervention demonstrates a reasonable level of continuity.

In the out-of-hours domain, relationship continuity may not be present. The feasibility of primary care physicians providing 24-hour care is influenced by several factors, including provider preferences, patient needs, existing market supply, and financial considerations (O’Malley *et al*., 2012). Therefore, if OOH-PC is not provided by the usual physician, a mechanism is necessary to facilitate the sharing of health information and systematic notification procedures to maintain information continuity between providers to prevent fragmentation of care (O’Malley *et al*., 2012). However, the exchange of patient information is of interest for all healthcare stakeholders and interventions, especially in the context of integrated care. Interaction is needed, for example, for a scheduled follow-up with the usual primary physician or when complications and/or exacerbations of previously treated conditions arise (O’Malley *et al*., 2012). The degree to which primary care interventions and modalities provide continuity of relationship and care should be monitored, assessed, and included in economic evaluations.

Incorporating continuity of care into economic evaluations is not common. Nonetheless, an Australian study assessed continuity of care as a qualitative aspect of its economic evaluation (KPMG, 2018). The study evaluated changes in continuity of care after the implementation of a primary care nurse-led clinic with services offered by a nurse and a collaborating GP, who visited the site bi-weekly (KPMG, 2018). This study revealed improved continuity of care by allowing the community to follow up on health-related issues before seeing a specialist and by acting as a link between the community and other health service providers in the wider region (KPMG, 2018).

Several measures of continuity of care exist, including the Usual Provider of Care index, the Bice-Boxerman Continuity of Care index, the Herfindahl Index, and the Sequential Continuity of Care Index (Pollack *et al*., 2016). Additionally, the measurement of coordination between professionals of different disciplines could use tools such as the relational coordination survey (Gittell, 2011).

## Discussion and research implications

Various economic evaluation techniques can determine the value-for-money of OOH-PC while including the three proposed additional effects. Cost-effectiveness analyses (CEA), which measures effects in natural units, has the potential to individually incorporate the three proposed effect measures. Continuity of care, for instance, could be assessed using natural measures such as the change in the proportion of sequential patient visits at the same provider (Roos *et al*., 1998). It can also be assessed by changes in interprofessional team communication and relationship scores (Hustoft *et al*., 2018). The use of monetary CBA, which converts all health-related effects to monetary terms (Drummond *et al*., 2015), could incorporate all three additional effect measures. To monetise continuity of care, one can assign a value to its anticipated outcomes, such as avoided hospital use or prompt treatment initiations. However, it is important to avoid the potential issue of double counting when considering these outcomes. Moreover, CBA could potentially be more time-consuming than CEA and is criticised for assigning monetary values to health states (Tsiachristas *et al*., 2016). Cost–utility analysis can incorporate all additional measures. By considering health gains through health outcomes such as mortality, the inclusion of continuity of care is feasible (Tsiachristas *et al*., 2016). However, as with CBA, caution is needed to prevent double counting. A cost-consequence analysis (CCA) presents a range of outcomes alongside costs; therefore, it could integrate all the proposed additional effects. Due to its clarity, CCA is used to inform decision-making (Mauskopf *et al*., 1998) but is criticised for its inability to rank alternative interventions based on their effectiveness (Perkins *et al*., 2015). Multi-criteria decision analysis (MCDA) is a systematic comparison of different alternatives by considering multidimensional factors (Baran-Kooiker *et al*., 2018). MCDA could potentially incorporate all proposed effect measures for the evaluation of OOH-PC. However, MCDA presents the challenge of assigning weights to effects based on the preferences of stakeholders within a specific setting (Marsh *et al*., 2018). It is necessary to further explore the applicability of MCDA approaches to OOH-PC.

Productivity costs often have a strong impact on cost-effectiveness outcomes (Krol & Brouwer, 2014). Therefore, whether and how to include them in economic evaluation is a debate that has been ongoing for several years (Krol *et al*., 2013). Some argue that the inclusion of productivity costs raises equity concerns, as interventions aimed at the employed produce more favourable cost-effectiveness outputs compared to interventions aimed at the unemployed (Lensberg *et al*., 2013). However, excluding productivity costs due to equity concerns is contested because other cost types, such as medical costs, also discriminate across different population groups, such as between the young and the old (Krol *et al*., 2013). To accommodate equity concerns, it has been suggested to report productivity gains in non-monetary units such as days or hours gained or lost (Drummond *et al*., 2015). Additionally, equity concerns can be addressed by evaluating productivity gains or losses for the unemployed using shadow prices that consider the opportunity costs associated with unpaid work activities, including household work, shopping, and childcare (Drummond *et al*., 2015). On the other hand, there is a lack of standardisation and consensus regarding the methodology for measuring productivity costs (Jiang *et al*., 2022; Krol & Brouwer, 2014). Recently, recommendations considered the use of instruments that include both paid-work productivity losses and those related to unpaid work (Krol & Brouwer, 2014). These include the iMTA Productivity Cost Questionnaire (iPCQ) or the Valuation of Lost Productivity (Krol & Brouwer, 2014). The omission of productivity costs in economic evaluations may partly be due to some national health economic guidelines that prescribe a health system perspective (Jiang *et al*., 2022). However, many economic evaluations taken from a societal perspective still exclude productivity costs (Jiang *et al*., 2022; Krol *et al*., 2016). This suggests a potential bias in the selection of cost types, and decision-makers need to be mindful of their inclusion or exclusion whenever the perspective is (partially) societal (Krol *et al*., 2013). However, decision-makers should also be mindful of whether health-related quality of life (HRQoL) has already been factored in, as it accounts for the effects of productivity gains or losses on an individual (Jiang *et al*., 2022). Including both HRQoL and productivity gains or losses may result in duplicate counting (Jiang *et al*., 2022). In response to the debates surrounding the inclusion of productivity gains or losses, there is a suggestion to present two scenarios of cost-effectiveness results, one with and another without productivity (Pritchard & Sculpher, 2000).

The implementation of OOH-PC on a large scale may face challenges due to shortages in the health workforce (Velgan *et al*., 2023). These shortages already make it difficult to recruit healthcare providers to perform regular contractual hours, let alone out-of-hours (The Scottish Government, 2015). Because of these shortages, a trade-off between regular and out-of-hours is likely. The provision of OOH-PC can, on the one hand, attract healthcare providers for higher pay (Broadway *et al*., 2017; Longden *et al*., 2018), leaving a gap in regular-hour care. On the other hand, OOH-PC may not interest all providers due to, for example, the impact on their work–life balance (The Scottish Government, 2015). Whether OOH-PC will threaten the sustainability of regular-hour primary care practices remains uncertain. Therefore, the aim of improving access to regular-hour primary care while concurrently improving out-of-hour continuity of care through OOH-PC requires careful balancing and consideration in future research.

Due to the unscheduled nature of OOH-PC, there is a great diversity of care provided compared to regular-hour primary care (NHS: Health Education England, n.d.). As a result, there is a growing need for adapted training and career development to ensure that providers have the right skills to handle the increasingly challenging and complex environment (GP Training: Urgent and Unscheduled Care (Including Out-of-Hours), 2022). These skills include the ability to handle medical, surgical, and psychiatric emergencies out-of-hours, the ability to make appropriate referrals to hospitals and other professionals, to manage personal time and stress, and to maintain personal security and awareness of environmental security risks (Royal College of General Practitioners, 2019; GP Training: Urgent and Unscheduled Care (Including Out-of-Hours), 2022). Additionally, providers need to be informed about the prevailing governance approaches due to the existing links between OOH-PC, ambulance services, and EDs (Royal College of General Practitioners, 2019). Consequently, there is a recommendation for multidisciplinary teams for out-of-hours care (Royal College of General Practitioners, 2019). Therefore, it is essential that resource planning for the primary care workforce considers the degree of training needed to deliver OOH-PC effectively. However, additional training resources may not always be available, which can negatively affect OOH-PC provision.

While OOH-PC serves as a viable alternative to ED care, it has the potential to trigger a supply-induced surge in healthcare utilisation during non-office hours (Longden *et al*., 2018). This influx of patients outside of regular office hours may result in increased demand for healthcare resources, which can lead to additional healthcare expenses from the healthcare payers’ perspective (Morreel *et al*., 2022). Moreover, the provision of OOH-PC may not always be a feasible option for healthcare providers due to factors such as higher clinical indemnity insurance costs. In the United Kingdom, for instance, providers face elevated indemnity insurance expenses for out-of-hours services compared to their regular office hours counterparts (NHS England and the Department of Health, 2016).

During the identification of innovative measures for the evaluation of OOH-PC, methodological challenges were encountered. First, given our scope on economic evaluations of OOH-PC, we did not conduct a systematic search for studies that focused only on the effects of OOH-PC, nor for studies that examined economic evaluations of integrated care. However, we made a conscious effort to prioritise the scientific relevance of the sources that we utilised and attempted to mitigate potential biases by using recent systematic reviews and by conducting a thorough snowballing of the references included in the selected contributions. The second challenge concerns the identification of additional effect measures in the current multidimensional and multi-objective framework, which often involves engagement with different stakeholders to develop standardised measures. However, the present study constitutes a tool that can effectively facilitate the establishment, development, and advancement of economic evaluation mechanisms for OOH-PC in line with integrated care initiatives.

The use of additional effect measures can present certain obstacles. Determining the suitability of potential measures for a given situation necessitates an evaluation of their significance. This empirical evaluation can be difficult and time-consuming, as it relies on a comprehensive assessment of previous context-specific evidence regarding potential impacts.

## Conclusion

In this paper, we discussed effect measures for conducting broad welfare-gain-driven economic evaluations of OOH-PC by drawing on experience from integrated care programmes. A focus on resource use measures can be too limiting in the OOH-PC domain, where a wide range of outcomes are relevant from the health system and patient perspectives. In this regard, we identified three relevant effects not yet considered in previous economic evaluations of OOH-PC. These are ‘productivity loss’, ‘health promotion and early intervention’, and ‘continuity of care’. This proposal of additional effects is neither comprehensive nor exhaustive but serves to highlight how to broaden the economic evaluation of OOH-PC by considering additional processes and patient outcomes related to the out-of-hours context. Determining what to include or exclude depends on the specific context, considering the evaluation perspective and the strength of existing evidence supporting the significance of an effect measure within that context.

## Supporting information

Phiri et al. supplementary material Phiri et al. supplementary material

## Supplementary material

To view supplementary material for this article, please visit https://doi.org/10.1017/S1463423624000318

## References

[ref1] Agha Z , Schapira RM and Maker AH (2002) Cost effectiveness of telemedicine for the delivery of outpatient pulmonary care to a rural population. Telemedicine Journal and E-Health: The Official Journal of the American Telemedicine Association 8, 281–291. 10.1089/15305620260353171 12419022

[ref2] Allen L , Cummings JR and Hockenberry JM (2021) The impact of urgent care centers on nonemergent emergency department visits. Health Services Research 56, 721–730. 10.1111/1475-6773.13631 33559261 PMC8313962

[ref3] Baker A (2001) Crossing the quality chasm: a new health system for the 21st century. BMJ 323, 1192. 10.1136/BMJ.323.7322.1192 25057539

[ref4] Baran-Kooiker A , Czech M and Kooiker C (2018) Multi-criteria decision analysis (MCDA) models in health technology assessment of orphan drugs—a systematic literature review. Next steps in methodology development? Frontiers in Public Health 6, 404746. 10.3389/FPUBH.2018.00287/BIBTEX PMC6197072 30374435

[ref5] Barnes K , Agostino J , Ceramidas D and Douglas K (2022) After-hours presentations to community-based primary care in the Australian Capital Territory. Australian Journal of Primary Health 28, 232–238. 10.1071/PY21261 35296376

[ref6] Baxter S , Johnson M , Chambers D , Sutton A , Goyder E and Booth A (2018) The effects of integrated care: a systematic review of UK and international evidence. BMC Health Services Research 18, 1–13. 10.1186/S12913-018-3161-3 29747651 PMC5946491

[ref7] Berchet C and Nader C (2016) The organisation of out-of-hours primary care in OECD countries. *OECD Health Working Papers*. 10.1787/5JLR3CZBQW23-EN

[ref8] Borsky A , Zhan C , Miller T , Ngo-Metzger Q , Bierman AS and Meyers D (2018) Few Americans receive all high-priority, appropriate clinical preventive services. Health Affairs (Project Hope) 37, 925–928. 10.1377/HLTHAFF.2017.1248 29863918

[ref10] Broadway B , Kalb G , Li J and Scott A (2017) Do financial incentives influence GPs’ decisions to do after-hours work? A discrete choice labour supply model. Health Economics 26, e52–e66. 10.1002/HEC.3476 28217847

[ref11] Broekman S , Van Gils-Van Rooij E , Meijboom B , De Bakker D and Yzermans C (2017) Do out-of-hours general practitioner services and emergency departments cost more by collaborating or by working separately? A cost analysis. Journal of Primary Health Care 9, 212–219. 10.1071/HC17015 29530174

[ref12] Brogan C , Pickard D , Gray A , Fairman S and Hill A (1998) The use of out of hours health services: a cross sectional survey. BMJ 316, 524. 10.1136/BMJ.316.7130.524 9501716 PMC2665637

[ref13] Byford S and Raftery J (1998) Perspectives in economic evaluation. BMJ 316, 1529–1530. 10.1136/BMJ.316.7143.1529 9582152 PMC1113167

[ref14] Carret MLV , Fassa AG and Kawachi I (2007) Demand for emergency health service: factors associated with inappropriate use. BMC Health Services Research 7, 1–9. 10.1186/1472-6963-7-131/TABLES/5 17705873 PMC2034385

[ref15] Chesteen SA , Warren SE and Woolley FR (1986) A comparison of family practice clinics and free-standing emergency centers: organizational characteristics, process of care, and patient satisfaction. The Journal of Family Practice 23, 377–382. 3760804

[ref16] Colliers A , Bartholomeeusen S , Remmen R , Coenen S , Michiels B , Bastiaens H , Van Royen P , Verhoeven V , Holmgren P , De Ruyck B and Philips H (2016) Improving Care and Research Electronic Data Trust Antwerp (iCAREdata): a research database of linked data on out-of-hours primary care. BMC Research Notes 9, 1–7. 10.1186/S13104-016-2055-X/FIGURES/1 27142361 PMC4855754

[ref17] Colliers A , Remmen R , Streffer ML , Michiels B , Bartholomeeusen S , Monsieurs KG , Goris J , Coenen S , Verhoeven V and Philips H (2017) Implementation of a general practitioner cooperative adjacent to the emergency department of a hospital increases the caseload for the GPC but not for the emergency department. Acta Clinica Belgica: International Journal of Clinical and Laboratory Medicine 72, 49–54. 10.1080/17843286.2016.1245936 27748165

[ref18] Culyer A , Chalkidou K , Teerawattananon Y and Santatiwongchai B (2018) Rival perspectives in health technology assessment and other economic evaluations for investing in global and national health. Who decides? Who pays? F1000Research 7, 7. 10.12688/F1000RESEARCH.13284.1 29904588 PMC5961761

[ref19] Deidda M , Geue C , Kreif N , Dundas R and McIntosh E (2019) A framework for conducting economic evaluations alongside natural experiments. Social Science & Medicine (1982) 220, 353. 10.1016/J.SOCSCIMED.2018.11.032 30513485 PMC6323352

[ref20] Dent RL (2010) The effect of telephone nurse triage on the appropriate use of the emergency department. Nursing Clinics of North America 45, 65–69. 10.1016/j.cnur.2009.10.003 20189544

[ref21] Derlet RW and Ledesma A (1999) How do prudent laypeople define an emergency medical condition? The Journal of Emergency Medicine 17, 413–418. 10.1016/S0736-4679(99)00014-1 10338230

[ref22] Drummond M , Sculpher M , Claxton K , Stoddart G and Torrance G (2015) Methods for the economic evaluation of health care programmes (4th ed.). Oxford: Oxford University Press. https://books.google.co.uk/books?id=lvWACgAAQBAJ

[ref23] Durand AC , Gentile S , Devictor B , Palazzolo S , Vignally P , Gerbeaux P and Sambuc R (2011) ED patients: how nonurgent are they? Systematic review of the emergency medicine literature. The American Journal of Emergency Medicine 29, 333–345. 10.1016/J.AJEM.2010.01.003 20825838

[ref24] Eichler K , Imhof D , Chmiel C , Zoller M , Senn O , Rosemann T and Huber CA (2010) The provision of out-of-hours care and associated costs in an urban area of Switzerland: a cost description study. BMC Family Practice 11, 99. 10.1186/1471-2296-11-99 21171989 PMC3013078

[ref25] Evers S , Goossens M , de Vet H , van Tulder M and Ament A (2005) Criteria list for assessment of methodological quality of economic evaluations: consensus on health economic criteria. International Journal of Technology Assessment in Health Care 21, 240–245. http://www.beoz.unimaas.nl/chec/ 15921065

[ref26] Flaherty KE , Klarman MB , Cajusma Y , Schon J , Exantus L , Beau de Rochars VM , Baril C , Becker TK and Nelson EJ (2022) A nighttime telemedicine and medication delivery service to avert pediatric emergencies in Haiti: an exploratory cost-effectiveness analysis. The American Journal of Tropical Medicine and Hygiene 106, 1063. 10.4269/AJTMH.21-1068 35189597 PMC8991343

[ref27] Flynn DM (1998) Telephone triage as a strategy to ensure 24-hour access to medical care after the closure of supporting medical activity. Military Medicine 163, 702–706. 9795548

[ref28] Foster H , Moffat KR , Burns N , Gannon M , Macdonald S and O’donnell CA (2020) What do we know about demand, use and outcomes in primary care out-of-hours services? A systematic scoping review of international literature. BMJ Open 10, e033481. 10.1136/BMJOPEN-2019-033481 PMC7045150 31959608

[ref29] Friedberg MW , Hussey PS and Schneider EC (2010) Primary care: a critical review of the evidence on quality and costs of health care. Health Affairs 29, 766–772. 10.1377/HLTHAFF.2010.0025 20439859

[ref30] Fry MM (2011) A systematic review of the impact of afterhours care models on emergency departments, ambulance and general practice services. Australasian Emergency Nursing Journal 14, 217–225. 10.1016/J.AENJ.2011.09.001

[ref31] Garratt AM , Danielsen K and Hunskaar S (2007) Patient satisfaction questionnaires for primary care out-of-hours services: a systematic review. The British Journal of General Practice 57, 741. PMCID: PMC2151790 17761062 PMC2151790

[ref32] Gittell JH (2011) Organizing work to support relational co-ordination. International Journal of Human Resource Management 11, 517–539. 10.1080/095851900339747

[ref33] GP Training: Urgent and Unscheduled Care (Including Out-of-Hours) (2022) https://www.bmj.com/careers/article/gp-training-urgent-and-unscheduled-care-including-out-of-hours-

[ref34] Gray DJP , Sidaway-Lee K , White E , Thorne A and Evans PH (2018) Continuity of care with doctors—a matter of life and death? A systematic review of continuity of care and mortality. BMJ Open 8, e021161. 10.1136/BMJOPEN-2017-021161 PMC6042583 29959146

[ref35] Gulliford M , Naithani S and Morgan M (2006) What is “continuity of care”? Journal of Health Services Research & Policy 11, 248–250. 10.1258/135581906778476490 17018200

[ref36] Guttmann A , Schull MJ , Vermeulen MJ and Stukel TA (2011) Association between waiting times and short term mortality and hospital admission after departure from emergency department: population based cohort study from Ontario, Canada. BMJ 342, 6. 10.1136/BMJ.D2983 PMC3106148 21632665

[ref37] Handley N and Hollander JE (1999) Opportunity cost: the hidden toll of seeking health care. Health Affairs Blog. 10.1377/hblog20190429.592190

[ref38] Hansen BL and Munck A (1998) Out-of-hours service in Denmark: the effect of a structural change. The British Journal of General Practice 48, 1497–1499. 10024709 PMC1313198

[ref39] Hill AP and Freeman GK (2011) Promoting continuity of care in general practice. https://www.rcgp.org.uk/getmedia/bda9fb5b-e52b-454d-af1f-e27f3df4100f/RCGP-Continuity-of-Care.pdf

[ref40] Hong M , Thind A , Zaric GS and Sarma S (2020) The impact of improved access to after-hours primary care on emergency department and primary care utilization: a systematic review. Health Policy 124, 812–818. 10.1016/J.HEALTHPOL.2020.05.015 32513447

[ref41] Hostetter J , Schwarz N , Klug M , Wynne J and Basson MD (2020) Primary care visits increase utilization of evidence-based preventative health measures. BMC Family Practice 21, 1–10. 10.1186/S12875-020-01216-8/TABLES/3 32718313 PMC7385977

[ref42] Huibers LA , Moth G , Bondevik GT , Kersnik J , Huber CA , Christensen MB , Leutgeb R , Casado AM , Remmen R and Wensing M (2011) Diagnostic scope in out-of-hours primary care services in eight European countries: an observational study. BMC Family Practice 12, 30. 10.1186/1471-2296-12-30 21569483 PMC3114765

[ref43] Huibers L , Smits M , Renaud V , Giesen P and Wensing M (2011) Safety of telephone triage in out-of-hours care: a systematic review. Scandinavian Journal of Primary Health Care 29, 198–209. 10.3109/02813432.2011.629150 22126218 PMC3308461

[ref44] Hustoft M , Biringer E , Gjesdal S , Aßus J and Hetlevik Ø (2018) Relational coordination in interprofessional teams and its effect on patient-reported benefit and continuity of care: a prospective cohort study from rehabilitation centres in Western Norway. BMC Health Services Research 18, 1–9. 10.1186/S12913-018-3536-5/TABLES/4 30223847 PMC6142375

[ref45] Institute of Medicine (1997) Schools and health: our nation’s investment. Washington, DC: The National Academies Press. 10.17226/5153 25121262

[ref46] Jiang S , Wang Y , Si L , Zang X , Gu YY , Jiang Y , Liu GG and Wu J (2022) Incorporating productivity loss in health economic evaluations: a review of guidelines and practices worldwide for research agenda in China. BMJ Global Health 7, e009777. 10.1136/BMJGH-2022-009777 PMC9389102 35977755

[ref47] Keizer E , Smits M , Peters Y , Huibers L , Giesen P and Wensing M (2015) Contacts with out-of-hours primary care for nonurgent problems: patients’ beliefs or deficiencies in healthcare? Knowledge, attitudes, behaviors, education, and communication. BMC Medical Research Methodology 15, 1–8. 10.1186/S12875-015-0376-9/TABLES/4 26510620 PMC4625560

[ref48] Kontopantelis E , Reeves D , Valderas JM , Campbell S and Doran T (2013) Recorded quality of primary care for patients with diabetes in England before and after the introduction of a financial incentive scheme: a longitudinal observational study. BMJ Quality & Safety 22, 53–64. 10.1136/BMJQS-2012-001033 22918988

[ref49] Kornguth ML (1990) School illnesses: who’s absent and why? Pediatric Nursing 16, 95–99. https://europepmc.org/article/med/2359635 2359635

[ref50] KPMG (2018) Cost benefit analysis of nurse practitioners models of care. https://www.health.gov.au/sites/default/files/documents/2021/03/cost-benefit-analysis-of-nurse-practitioner-models-of-care.pdf

[ref51] Kraaijvanger N , Rijpsma D , Van Leeuwen H , Van Dijk N and Edwards M (2016) Self-referrals in a Dutch emergency department: how appropriate are they? European Journal of Emergency Medicine 23, 194–202. 10.1097/MEJ.0000000000000216 25380319

[ref52] Krol M and Brouwer W (2014) How to estimate productivity costs in economic evaluations. PharmacoEconomics 32, 335–344. 10.1007/S40273-014-0132-3 24504850

[ref53] Krol M , Brouwer W and Rutten F (2013) Productivity costs in economic evaluations: past, present, future. PharmacoEconomics 31, 537–549. 10.1007/S40273-013-0056-3/METRICS 23620213

[ref54] Krol M , Papenburg J , Tan SS , Brouwer W and Hakkaart L (2016) A noticeable difference? Productivity costs related to paid and unpaid work in economic evaluations on expensive drugs. The European Journal of Health Economics 17, 391. 10.1007/S10198-015-0685-X 25876834 PMC4837201

[ref55] Kubes JN , Graetz I , Wiley Z , Franks N and Kulshreshtha A (2021) Associations of telemedicine vs. in-person ambulatory visits and cancellation rates and 30-day follow-up hospitalizations and emergency department visits. Preventive Medicine Reports 24, 101629. 10.1016/J.PMEDR.2021.101629 34976682 PMC8684024

[ref56] Lattimer V , Sassi F , George S , Moore M , Turnbull J , Mullee M and Smith H (2000) Cost analysis of nurse telephone consultation in out of hours primary care: evidence from a randomised controlled trial. BMJ (Clinical Research Ed.) 320, 1053–1057. 10.1136/bmj.320.7241.1053 PMC27349 10764368

[ref57] Leibowitz R , Day S and Dunt D (2003) A systematic review of the effect of different models of after-hours primary medical care services on clinical outcome, medical workload, and patient and GP satisfaction. Family Practice 20, 311–317. 10.1093/FAMPRA/CMG313 12738701

[ref58] Lensberg BR , Drummond MF , Danchenko N , Despiégel N and François C (2013) Challenges in measuring and valuing productivity costs, and their relevance in mood disorders. ClinicoEconomics and Outcomes Research: CEOR 5, 565. 10.2147/CEOR.S44866 24273412 PMC3836685

[ref59] Levine S , Malone E , Lekiachvili A and Briss P (2019) Health care industry insights: why the use of preventive services is still low. Preventing Chronic Disease 16, E30. 10.5888/PCD16.180625 30873937 PMC6429690

[ref60] Lin CP , Loy S , Boothe WD , Bennett K , Tarbox MB , Prabhu F and Sturgeon A (2021) Value of dermatology nights at a student-run free clinic. Proceedings (Baylor University. Medical Center) 34, 260. 10.1080/08998280.2020.1834771 PMC7901410 33678959

[ref61] Liu F , Guo P , Wang Y and Xi Y (n.d.) Improving health outcomes with less cost? Provision of mobile clinic in developing economies. Retrieved 19 April 2023 from https://ssrn.com/abstract=4220391

[ref62] Longden T , Hall J and van Gool K (2018) Supplier-induced demand for urgent after-hours primary care services. Health Economics 27, 1594–1608. 10.1002/HEC.3779 29781557

[ref63] Lowe RA , Localio AR , Schwarz DF , Williams S , Tuton LW , Maroney S , Nicklin D , Goldfarb N , Vojta DD and Feldman HI (2005) Association between primary care practice characteristics and emergency department use in a medicaid managed care organization. Medical Care 43, 792–800. 10.1097/01.MLR.0000170413.60054.54 16034293

[ref64] Maciosek MV , LaFrance AB , Dehmer SP , McGree DA , Flottemesch TJ , Xu Z and Solberg LI (2017) Updated priorities among effective clinical preventive services. Annals of Family Medicine 15, 14. 10.1370/AFM.2017 28376457 PMC5217840

[ref65] Marsh K , Thokala P , Youngkong S and Chalkidou K (2018) Incorporating MCDA into HTA: challenges and potential solutions, with a focus on lower income settings. Cost Effectiveness and Resource Allocation: C/E 16(Suppl 1), 1–19. 10.1186/S12962-018-0125-8 30455602 PMC6225551

[ref66] Mauskopf JA , Paul JE , Grant DM and Stergachis A (1998) The role of cost-consequence analysis in healthcare decision-making. PharmacoEconomics 13, 277–288. 10.2165/00019053-199813030-00002 10178653

[ref67] Measures Management Systems (2023) Type of measures. https://mmshub.cms.gov/about-quality/new-to-measures/types

[ref68] Moe J , Oland R and Moe G (2019) Impact of a primary care after-hours clinic on avoidable emergency department visits and costs. Healthcare Quarterly 22, 42–47. 10.12927/HCQ.2019.25837 31244467

[ref69] Mohsin M , Forero R , Ieraci S , Bauman AE , Young L and Santiano N (2007) A population follow-up study of patients who left an emergency department without being seen by a medical officer. Emergency Medicine Journal: EMJ 24, 175–179. 10.1136/EMJ.2006.038679 17351221 PMC2660023

[ref70] Moore S , Young T , Irving A , Goodacre S , Brennan A and Amos Y (2021) Controlled observational study and economic evaluation of the effect of city-centre night-time alcohol intoxication management services on the emergency care system compared with usual care. Emergency Medicine Journal 38, 504–510. 10.1136/EMERMED-2019-209273 33148772

[ref71] Morreel S , Homburg I , Philips H , De Graeve D , Monsieurs KG , Meysman J , Lefevere E and Verhoeven V (2022) Cost effects of nurse led triage at an emergency department with the advice to consult the adjacent general practice cooperative for low-risk patients, a cluster randomised trial. Health Policy (Amsterdam, Netherlands) 126, 980–987. 10.1016/j.healthpol.2022.08.002 35963797

[ref72] Moth G , Huibers L and Vedsted P (2013) From doctor to nurse triage in the Danish out-of-hours primary care service: simulated effects on costs. International Journal of Family Medicine 2013, 987834. 10.1155/2013/987834 24194984 PMC3806230

[ref73] NHS England and the Department of Health (2016) GP indemnity review. https://www.england.nhs.uk/wp-content/uploads/2016/07/gp-indemnity-rev-summary.pdf

[ref74] NHS: Health Education England (n.d.) Out of hours GP training: guidance for GP trainees. Retrieved 29 June 2023 from https://heeoe.hee.nhs.uk/sites/default/files/heeoe_ooh_training_guidance_for_trainees.pdf

[ref75] NHS Primary Care Commissioning (2012) Improving GP access and responsiveness: productive primary care. https://www.yumpu.com/en/document/read/5402594/improving-gp-access-and-responsiveness-productive-primary-care

[ref76] Nolte E and Pitchforth E (2014) What is the evidence on the economic impacts of integrated care? (Vol. 11). https://iris.who.int/bitstream/handle/10665/332002/Policy-summary-11-2077-1584-eng.pdf?sequence=1

[ref77] O’Donnell CA , Foster H , Macdonald S , Burns N and Gannon M (2015) Out-of-hours primary medical care: what can research tell us? Findings from a rapid systematic review and qualitative study. Scottish Government. http://www.gov.scot/Resource/0049/00492082.pdf

[ref78] O’Dowd A (2006) Cost of out of hours care was 22% higher than predicted in England. BMJ: British Medical Journal 332, 1113. 10.1136/BMJ.332.7550.1113-C PMC1459577 16690658

[ref79] O’Malley AS , Samuel D , Bond AM and Carrier E (2012) After-hours care and its coordination with primary care in the U.S. Journal of General Internal Medicine 27, 1406. 10.1007/S11606-012-2087-4 22653379 PMC3475839

[ref80] Oriol NE , Cote PJ , Vavasis AP , Bennet J , DeLorenzo D , Blanc P and Kohane I (2009) Calculating the return on investment of mobile healthcare. BMC Medicine 7, 1–6. 10.1186/1741-7015-7-27/COMMENTS 19490605 PMC2697174

[ref81] Patel KB , Turner K , Alishahi Tabriz A , Gonzalez BD , Oswald LB , Nguyen OT , Hong YR , Jim HSL , Nichols AC , Wang X , Robinson E , Naso C and Spiess PE (2023) Estimated indirect cost savings of using telehealth among nonelderly patients with cancer. JAMA Network Open 6, e2250211–e2250211. 10.1001/JAMANETWORKOPEN.2022.50211 36626174 PMC9856804

[ref82] Patwardhan A , Davis J , Murphy P and Ryan SF (2012) After-hours access of convenient care clinics and cost savings associated with avoidance of higher-cost sites of care. Journal of Primary Care & Community Health 3, 243–245. 10.1177/2150131911436251 23804168

[ref83] Perkins C , Steinbach R , Tompson L , Green J , Johnson S , Grundy C , Wilkinson P and Edwards P (2015) Cost–benefit analysis: methodological challenges of evaluating large-scale public health interventions and a worked example of the costs and benefits of part-night lighting. https://www.ncbi.nlm.nih.gov/books/NBK316508/

[ref84] Piehl MD , Clemens CJ and Joines JD (2000) “Narrowing the Gap”: decreasing emergency department use by children enrolled in the Medicaid program by improving access to primary care. Archives of Pediatrics & Adolescent Medicine 154, 791–795. 10.1001/ARCHPEDI.154.8.791 10922275

[ref85] Plochg T , Klazinga NS and Starfield B (2009) Transforming medical professionalism to fit changing health needs. BMC Medicine 7, 64. 10.1186/1741-7015-7-64 19857246 PMC2773806

[ref86] Pollack CE , Hussey PS , Rudin RS , Fox DS , Lai J and Schneider EC (2016) Measuring care continuity: a comparison of claims-based methods. Medical Care 54, e30. 10.1097/MLR.0000000000000018 24309664 PMC4101051

[ref87] Poole SR , Schmitt BD , Carruth T , Peterson-Smith A and Slusarski M (1993) After-hours telephone coverage: the application of an area-wide telephone triage and advice system for pediatric practices. Pediatrics 92, 670–679. 10.1542/peds.92.5.670 8414853

[ref88] Pritchard C and Sculpher M (2000) Productivity costs: principles and practice in economic evaluation: OHE. https://www.ohe.org/publications/productivity-costs-principles-and-practice-economic-evaluation/

[ref89] Radhakrishnan M (2017) Health promotion and disease prevention through population-based interventions, including action to address social determinants and health inequity. Journal of Nursing & Care 06, 5. 10.4172/2167-1168-C1-052

[ref90] Ray KN , Chari AV , Engberg J , Bertolet M and Mehrotra A (2015) Opportunity costs of ambulatory medical care in the United States. The American Journal of Managed Care 21, 567. PMCID: PMC8085714 26295356 PMC8085714

[ref91] Reuter P-G , Desmettre T , Guinemer S , Ducros O , Begey S , Ricard-Hibon A , Billier L , Grignon O , Megy-Michoux I , Latouff J-N , Sourbes A , Latier J , Durand-Zaleski I , Lapostolle F , Vicaut E and Adnet F (2016) Effectiveness and cost-effectiveness of telephone consultations for fever or gastroenteritis using a formalised procedure in general practice: study protocol of a cluster randomised controlled trial. Trials 17, 461. 10.1186/s13063-016-1585-9 27659897 PMC5034584

[ref92] Rocks S , Berntson D , Gil-Salmerón A , Kadu M , Ehrenberg N , Stein V and Tsiachristas A (2020) Cost and effects of integrated care: a systematic literature review and meta-analysis. The European Journal of Health Economics: HEPAC: Health Economics in Prevention and Care 21, 1211–1221. 10.1007/S10198-020-01217-5 32632820 PMC7561551

[ref93] Roos NP , Carrière KC and Friesen D (1998) Factors influencing the frequency of visits by hypertensive patients to primary care physicians in Winnipeg. CMAJ: Canadian Medical Association Journal 159, 777. PMCID: PMC1232734 9805023 PMC1232734

[ref94] Royal College of General Practitioners (2019) Out of hours care. https://www.rcgp.org.uk/representing-you/policy-areas/out-of-hours

[ref95] Schneider E , Burgers J , Friedberg M , Rosenthal MB , Leape L and Schneider E (2011) Defining and measuring integrated patient care: promoting the next frontier in health care delivery. Medical Care Research and Review: MCRR 68, 112–127. 10.1177/1077558710371485 20555018

[ref96] Scott A , Simoens S , Heaney D , O’Donnell CA , Thomson H , Moffat KJ , Ross S and Drummond N (2004) What does GP out of hours care cost? An analysis of different models of out of hours care in Scotland. Scottish Medical Journal 49, 61–66. 10.1177/003693300404900208 15209145

[ref97] Shaw S , Rosen R and Rumbold B (2011) What is integrated care? https://www.nuffieldtrust.org.uk/research/what-is-integrated-care

[ref98] Sidaway-Lee K , Gray DP and Evans P (2019) A method for measuring continuity of care in day-to-day general practice: a quantitative analysis of appointment data. The British Journal of General Practice 69, e356. 10.3399/BJGP19X701813 30803982 PMC6478463

[ref99] Smits M , Keizer E , Huibers L and Giesen P (2014) GPs’ experiences with out-of-hours GP cooperatives: a survey study from the Netherlands. The European Journal of General Practice 20, 196–201. 10.3109/13814788.2013.839652 24160262

[ref100] Snoswell CL , Taylor ML , Comans TA , Smith AC , Gray LC and Caffery LJ (2020) Determining if telehealth can reduce health system costs: scoping review. Journal of Medical Internet Research 22, e17298. 10.2196/17298 33074157 PMC7605980

[ref101] Starfield B , Shi L and Macinko J (2005) Contribution of primary care to health systems and health. The Milbank Quarterly 83, 457–502. 10.1111/J.1468-0009.2005.00409.X 16202000 PMC2690145

[ref102] Steeman L , Uijen M , Plat E , Huibers L , Smits M and Giesen P (2020) Out-of-hours primary care in 26 European countries: an overview of organizational models. Family Practice 37, 744–750. 10.1093/FAMPRA/CMAA064 32597962 PMC7699311

[ref103] Sterner SE , Coco T , Monroe KW , King WD and Losek JD (2012) A new after-hours clinic model provides cost-saving, faster care compared with a pediatric emergency department. Pediatric Emergency Care 28, 1162–1165. 10.1097/PEC.0b013e318271733e 23114241

[ref104] Steuten L , Vrijhoef B , Severens H , Van Merode F and Spreeuwenberg C (2006) Are we measuring what matters in health technology assessment of disease management? Systematic literature review. International Journal of Technology Assessment in Health Care 22, 47–57. 10.1017/S0266462306050835 16673680

[ref105] Stillmank E , Bloesl K , McArthur E , Artz B and Lancaster RJ (2019) A cost-benefit analysis of a community free clinic. Journal of Community Health Nursing 36, 91–101. 10.1080/07370016.2019.1583838 30990744

[ref106] Sun S , Lu SF and Rui H (2020) Does telemedicine reduce emergency room congestion? Evidence from New York State. Information Systems Research 31, 972–986. 10.1287/ISRE.2020.0926

[ref108] The Scottish Government (2015) Pulling together: transforming urgent care for the people of Scotland The Report of the Independent Review of Primary Care Out of Hours Services. http://www.gov.scot/topics/health/services/nrpcooh

[ref109] Tsiachristas A , Cramm JM , Nieboer A and Rutten-Van Mölken M (2013) Broader economic evaluation of disease management programs using multi-criteria decision analysis. International Journal of Technology Assessment in Health Care 29, 301–308. 10.1017/S0266462313000202 23759317

[ref110] Tsiachristas A , Stein KV , Evers S and Rutten-van Mölken M (2016) Performing economic evaluation of integrated care: highway to hell or stairway to heaven? International Journal of Integrated Care 16, 1–12. 10.5334/IJIC.2472 PMC5354211 28316543

[ref113] van Uden CJT , Ament AJHA , Voss GBWE , Wesseling G , Winkens RAG , van Schayck OCP and Crebolder HFJM (2006) Out-of-hours primary care. Implications of organisation on costs. BMC Family Practice 7, 29. 10.1186/1471-2296-7-29 16674814 PMC1464145

[ref114] Van Weel C and Kidd MR (2018) Why strengthening primary health care is essential to achieving universal health coverage. CMAJ: Canadian Medical Association Journal 190, E463. 10.1503/CMAJ.170784 29661815 PMC5903888

[ref115] Velgan M , Vanderheyde T , Kalda R and Michels N (2023) Driving forces of GPs’ migration in Europe: an exploratory qualitative study. BJGP Open 7, 2. 10.3399/BJGPO.2022.0132 PMC10354382 36717117

[ref116] Weinstein MC , Siegel JE , Gold MR , Kamlet MS and Russell LB (1996) Recommendations of the panel on cost-effectiveness in health and medicine. JAMA 276, 1253–1258. 10.1001/JAMA.1996.03540150055031 8849754

[ref117] Whittaker W , Anselmi L , Kristensen SR , Lau YS , Bailey S , Bower P , Checkland K , Elvey R , Rothwell K , Stokes J and Hodgson D (2016) Associations between extending access to primary care and emergency department visits: a difference-in-differences analysis. PLOS Medicine 13, e1002113. 10.1371/JOURNAL.PMED.1002113 27598248 PMC5012704

[ref118] WHO and UNICEF. (2022) Primary health care measurement framework and indicators: monitoring health systems through a primary health care lens. https://www.who.int/publications/i/item/9789240044210

[ref119] Wijers N , Schoonhoven L , Giesen P , Vrijhoef H , Van Der Burgt R , Mintjes J , Wensing M and Laurant M (2012) The effectiveness of nurse practitioners working at a GP cooperative: a study protocol. BMC Family Practice 13, 1–9. 10.1186/1471-2296-13-75 22870898 PMC3503817

[ref120] World Health Organization (2018) Building the economic case for primary health care: a scoping review. https://www.who.int/docs/default-source/primary-health-care-conference/phc---economic-case.pdf

[ref121] Xu KT (2002) Usual source of care in preventive service use: a regular doctor versus a regular site. Health Services Research 37, 1509–1529. 10.1111/1475-6773.10524 12546284 PMC1464041

[ref122] Zhang W , Cheng B , Zhu W , Huang X and Shen C (2021) Effect of telemedicine on quality of care in patients with coexisting hypertension and diabetes: a systematic review and meta-analysis. Telemedicine Journal and E-Health: The Official Journal of the American Telemedicine Association 27, 603–614. 10.1089/TMJ.2020.0122 32976084

[ref123] Zhou Y , Abel G , Warren F , Roland M , Campbell J and Lyratzopoulos G (2015) Do difficulties in accessing in-hours primary care predict higher use of out-of-hours GP services? Evidence from an English National Patient Survey. Emergency Medicine Journal 32, 373–378. 10.1136/EMERMED-2013-203451 24850778 PMC4413677

