# Supplementary material for: The need for a broad perspective when assessing value-for-money for out-of-hours primary care
Source: Prim Health Care Res Dev. 2024 Sep 20;25:e37. doi: 10.1017/S1463423624000318 (PMC11464846; doi:10.1017/S1463423624000318)
Supplement: Phiri et al. supplementary material [file S1463423624000318sup001.pdf]

Appendix A: Systematic Literature Search Strategy

We systematically identified publications in PubMed, SCOPUS, Web of Science, EconLit, Cochrane reviews, NHSEED, and Health Technology Assessment databases based on title/abstract screening (and full-text if necessary) to retrieve topics and general specifications of OOH-PC studies. On July 13, 2023, search queries (see tables A1-A7) were generated based on exploratory searches and on the definitions of two key concepts: 'OOH-PC' and 'economic evaluation'. OOH-PC is defined as primary care services that are available outside of regular business hours, specifically from 6:00 PM to 8:00 AM on weekdays and during weekends and public holidays (Søvsø et al., 2019). On the other hand, economic evaluations refer to comparative analyses of alternative courses of action in terms of both their costs and consequences (Drummond et al., 2015).

Search Strategies

Table A1: PubMed Search Strategy

**Search Number**	**Search Terms**	**Number of records**
#1	"out of hour*"[Title/Abstract] OR "out of hour*"[Title/Abstract] OR "after hour*"[Title/Abstract] OR "after hour*"[Title/Abstract] OR "extended hour*"[All Fields] OR "extended hour*"[Title/Abstract] OR "week end*"[Title/Abstract] OR "weekend*"[Title/Abstract] OR "evening*"[Title/Abstract] OR "out of office hours*"[Title/Abstract] OR "outside office hour*"[Title/Abstract] OR "outside normal working hour*"[Title/Abstract] OR "holiday*"[Title/Abstract] OR “night”[Title/Abstract] OR "After-Hours Care"[MeSH Terms] OR "Night Care"[MeSH Terms]	117,759
#2	"appointment*"[Title/Abstract] OR "clinic"[Title/Abstract] OR "clinics"[Title/Abstract] OR "center"[Title/Abstract] OR "centers"[Title/Abstract] OR "centre"[Title/Abstract] OR "centres"[Title/Abstract] OR "care"[Title/Abstract] OR "unit"[Title/Abstract] OR "units"[Title/Abstract] OR "service"[Title/Abstract] OR "services"[Title/Abstract] OR "facility"[Title/Abstract] OR "facilities"[Title/Abstract] OR "healthcare"[Title/Abstract] OR "primary care"[Title/Abstract] OR "nurse led"[Title/Abstract] OR "physician assistant*"[Title/Abstract] OR "GP led"[Title/Abstract] OR "GP practice"[Title] OR "general practioners"[Title/Abstract] OR "general practioner*"[Title/Abstract] OR "visit*"[Title/Abstract] OR "walk in*"[Title/Abstract] OR "gp cooperative"[Title/Abstract] OR "deputizing service"[Title/Abstract] OR "deputising services"[Title/Abstract] OR "rota group"[Title/Abstract]	4117,840
#3	"economic*"[Title/Abstract] OR "economic evaluation*"[Title/Abstract] OR "economic analy*"[Title/Abstract] OR "cost analy*"[Title/Abstract] OR "cost effectiveness"[Title/Abstract] OR "cost benefit*"[Title/Abstract] OR "cost utilit*"[Title/Abstract] OR "return on investment*"[Title/Abstract] OR "cost consequence*"[Title/Abstract] OR "multi criteria decision analy*"[Title/Abstract] OR "value-for-money"[Title/Abstract] OR "value-for-money"[Title/Abstract] OR "costs and cost analysis"[MeSH Terms]	643,435
#4	#1 AND #2 AND #3	1294

Table A2: Cochrane Library Search Strategy

Search Number	Search Terms	Number of records
#1	("out-of-hour" or "out-of-hours" or "out of hour" or "out of hours" or weekend* or "week ends" or "week end" or week-end* or evening* or after-hour* or "after hour" or "after hours" or "extended hours" or "extended hour" or extended-hour* or evening* or "out of office hours" or "out of office hour" or "outside office hours" or "outside office hour" or "outside normal working hours" or "outside normal working hour" or holiday* or night*):ti,ab	35,336
#2	(appointment* or clinic* or care or healthcare or consultation* or center* or centre* or care or healthcare or unit* or facility or facilities or "primary care" or "rota group" or "rota groups" or visit* or "general practitioners" or "general practitioner" or "gp cooperatives" or "gp cooperative" or "deputising service" or "deputising services" or "deputizing service" or "deputizing service" or "walk-in" or "urgent care" or service* or "gp led" or "nurse led"):ti,ab	962,726
#3	(economic* or "economic evaluation" or "economic analysis" or "economic analyses" or "cost analysis" or "cost analyses" or "cost effectiveness" or "cost-effectiveness" or "cost benefit" or "cost-benefit" or "cost utility" or "cost utilities" or "cost-utility" or "cost-utilities" or "return on investment" or "return on investments" or "cost consequence" or "cost consequence" or "cost-consequence" or "cost-consequences" or "multi-criteria decision analysis" or "multi-criteria decision analyses" or "value-for-money" or "value for money"):ti,ab,kw	42,072
#4	#1 AND #2 AND #3	501

Table A3: SCOPUS Search Strategy

Search Number	Search Terms	Number of records
#1	(TITLE-ABS("out-of-hour*" OR "out of hour*" OR "weekend*" OR "week end" OR "after hour*" OR "after-hour*" OR "extended hour*" OR "extended-hour*" OR "evening" OR "out of office hour*" OR "outside office hour*" OR "outside normal working hour*" OR "holiday*" or "night*") NEAR/5 ("appointment*" OR "clinic*" OR "care" OR "healthcare" OR "consultation*" OR "center" OR "centre" OR "unit" OR "facility" or "facilities" OR "primarycare" OR "rota group*" OR "visit*" OR "general practitioner*" OR "gp cooperative*" OR "deputising service*" or "deputizing service*" OR "walk-in" OR "service")) AND (TITLE-ABS(economic* or "economic evaluation" or "economic analysis" or "economic analyses" or "cost analysis" or "cost analyses" or "cost effectiveness" or "cost-effectiveness" or "cost benefit" or "cost-benefit" or "cost utility" or "cost utilities" or "cost-utility" or "cost-utilities" or "return on investment" or "return on investments" or "cost consequence" or "cost consequence" or "cost-consequence" or "cost-consequences" or "multi-criteria decision analysis" or "multi-criteria decision analyses" or "value-for-money" or "value for money"))	479

Table A4: Web of Science Search Strategy

Search Number	Search Terms	Number of records
#1	TI=(("out-of-hour*" OR "out of hour*" OR "weekend*" OR "week end" OR "after hour*" OR "after-hour*" OR "extended hour*" OR "extended-hour*" OR "evening" OR "out of office hour*" OR "outside office hour*" OR "outside normal working hour*" OR "holiday*" or "night*") NEAR/3 ("appointment*" OR "clinic*" OR "care" OR "healthcare" OR "consultation*" OR "center" OR "centre" OR "unit" OR "facility" or "facilities" OR "primarycare" OR "rota group*" OR "visit*" OR "general practitioner*" OR "gp cooperative*" OR "deputising service*" or "deputizing service*" OR "walk-in" OR "service"))	1804
#2	AB=(("out-of-hour*" OR "out of hour*" OR "weekend*" OR "week end" OR "after hour*" OR "after-hour*" OR "extended hour*" OR "extended-hour*" OR "evening" OR "out of office hour*" OR "outside office hour*" OR "outside normal working hour*" OR "holiday*" or "night*") NEAR/3 ("appointment*" OR "clinic*" OR "care" OR "healthcare" OR "consultation*" OR "center" OR "centre" OR "unit" OR "facility" or "facilities" OR "primarycare" OR "rota group*" OR "visit*" OR "general practitioner*" OR "gp cooperative*" OR "deputising service*" or "deputizing service*" OR "walk-in" OR "service"))	4253
#3	TI=(economic* or "economic evaluation" or "economic analysis" or "economic analyses" or "cost analysis" or "cost analyses" or "cost effectiveness" or "cost-effectiveness" or "cost benefit" or "cost-benefit" or "cost utility" or "cost utilities" or "cost-utility" or "cost-utilities" or "return on investment" or "return on investments" or "cost consequence" or "cost consequence" or "cost-consequence" or "cost-consequences" or "multi-criteria decision analysis" or "multi-criteria decision analyses" or "value-for-money" or "value for money")	402,417
#4	AB=(economic* or "economic evaluation" or "economic analysis" or "economic analyses" or "cost analysis" or "cost analyses" or "cost effectiveness" or "cost-effectiveness" or "cost benefit" or "cost-benefit" or "cost utility" or "cost utilities" or "cost-utility" or "cost-utilities" or "return on investment" or "return on investments" or "cost consequence" or "cost consequence" or "cost-consequence" or "cost-consequences" or "multi-criteria decision analysis" or "multi-criteria decision analyses" or "value-for-money" or "value for money")	1,248,621
#5	(#1 OR 2) AND (#3 OR #4)	279

Table A5: Economic Evaluation Database Search Strategy

Search Number	Search Terms	Number of records
#1	“out-of-hour” or “out-of-hours” or “out of hour” or “out of hours” or weekend* or “week ends” or “week end” or evening* or after-hour* or “after hour” or “after hour” or “after hours” or “extended hours” or “extended hour” or “extended-hour” or evening* or “out of office hours” or “out of office hour” or “outside office hours” or “outside office hour” or “outside normal working hours” or “outside working hour” or holiday* or night*	9

Table A6: EconLit Search Strategy

Search Number	Search Terms	Number of records
#1	(title("out-of-hour" OR "out-of-hours" OR "out of hour" OR "out of hours" OR weekend* OR "week ends" OR "week end" OR week-end* OR evening* OR after-hour* OR "after hour" OR "after hours" OR "extended hours" OR "extended hour" OR extended-hour* OR evening* OR "out of office hours" OR "out of office hour" OR "outside office hours" OR "outside office hour" OR "outside normal working hours" OR "outside normal working hour" OR holiday* OR night*) OR abstract("out-of-hour" OR "out-of-hours" OR "out of hour" OR "out of hours" OR weekend* OR "week ends" OR "week end" OR week-end* OR evening* OR after-hour* OR "after hour" OR "after hours" OR "extended hours" OR "extended hour" OR extended-hour* OR evening* OR "out of office hours" OR "out of office hour" OR "outside office hours" OR "outside office hour" OR "outside normal working hours" OR "outside normal working hour" OR holiday* OR night*)) AND (title(appointment* or clinic* or care or healthcare or consultation* or center* or centre* or care or healthcare or unit* or facility or facilities or "primary care" or "rota group" or "rota groups" or visit* or "general practitioners" or "general practitioner" or "gp cooperatives" or "gp cooperative" or "deputising service" or "deputising services" or "deputizing service" or "deputizing service" or "walk-in" or "urgent care" or service* or "gp led" or "nurse led") OR abstract (appointment* or clinic* or care or healthcare or consultation* or center* or centre* or care or healthcare or unit* or facility or facilities or "primary care" or "rota group" or "rota groups" or visit* or "general practitioners" or "general practitioner" or "gp cooperatives" or "gp cooperative" or "deputising service" or "deputising services" or "deputizing service" or "deputizing service" or "walk-in" or "urgent care" or service* or "gp led" or "nurse led"))	148

Table A7: Health Technology Assessment Search Strategy

Search Number	Search Terms	Number of records
#1	“out-of-hour” or “out-of-hours” or “out of hour” or “out of hours” or weekend* or “week ends” or “week end” or evening* or after-hour* or “after hour” or “after hour” or “after hours” or “extended hours” or “extended hour” or “extended-hour” or evening* or “out of office hours” or “out of office hour” or “outside office hours” or “outside office hour” or “outside normal working hours” or “outside working hour” or holiday* or night*	9

Appendix B: Systematic Literature Search Process (PRISMA Diagram)

The screening on title/abstract and full text if necessary was conducted by a single reviewer (JP), in consultation with co-authors in case of doubt. For each study that met the eligibility criteria, JP retrieved the study overview, comparators, and effect measures covered. Using the online databases, we identified 2717 articles, and one additional one was found through reviewing systematic review references. 2419 unique articles matching the search criteria listed were retained. Based on title, and abstract screening with predefined eligibility criteria, we excluded 2318 articles. We analysed full texts for 101 abstracts and excluded 88 of them. Finally, we obtained 13 studies performing a cost-effectiveness analysis of OOH-PC. The adapted PRISMA diagram of the screening process with inclusion and exclusion criteria is shown in Figure B1.

Figure B1: Adapted PRISMA flow diagram of the systematic search process on cost-effectiveness studies for OOH-PC

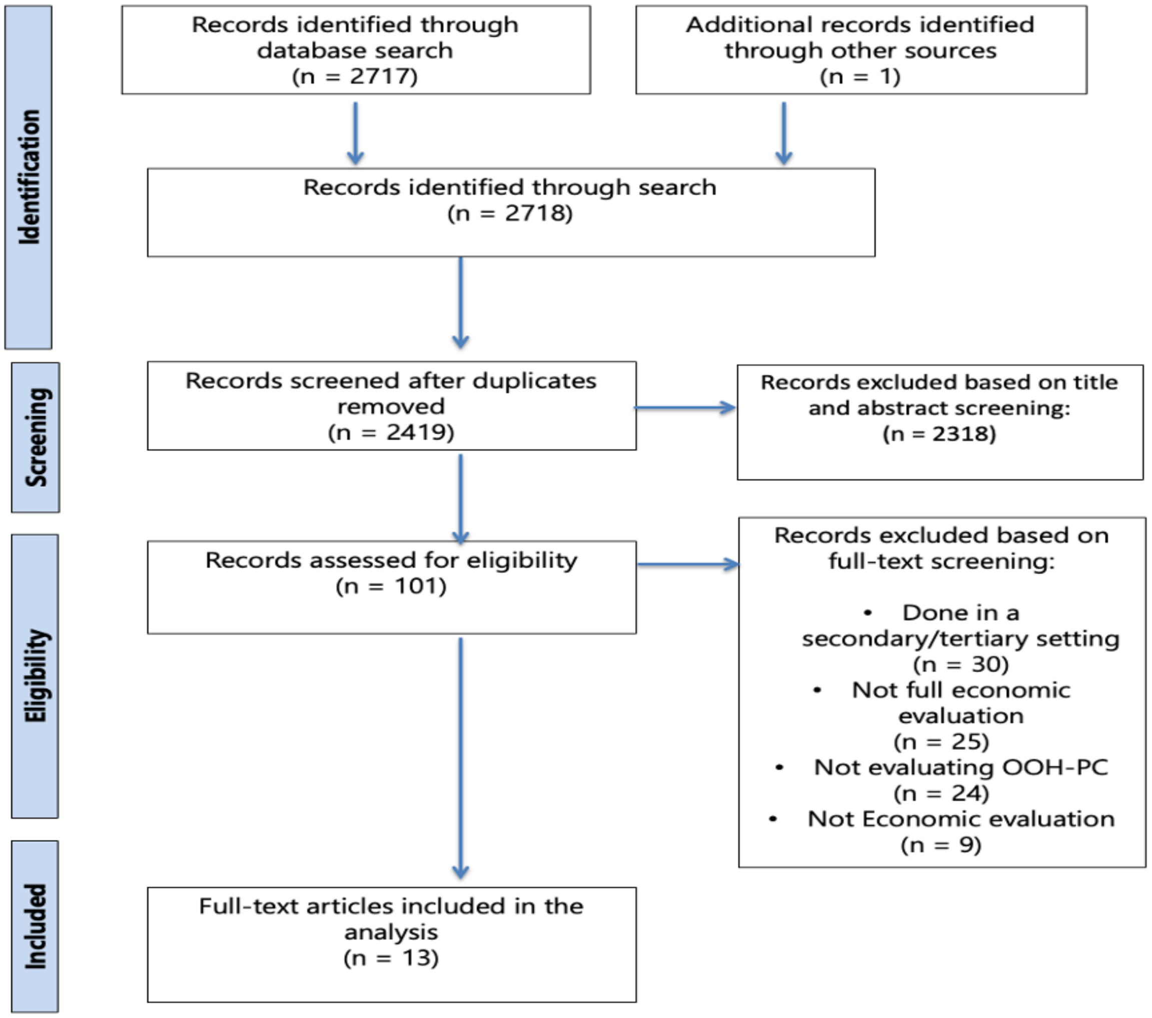

Appendix C: Quality Assessment

Each article was awarded 1 point for correctly addressing a Consensus Health Economic Criteria (CHEC) list question and 0 points if it did not address the question at all (Evers et al., 2005). For questions that were not applicable, no points were given. The total score was tallied out of 19 points, and a percentage was calculated by dividing the total score by the number of applicable questions. Based on previous publications that used the CHEC list, each study was then assigned a quality level (High ≥75%, Moderate 51–74%, Low ≤50%) (van Delft et al., 2023; van Mastrigt et al., 2016). Caution must be maintained with these levels, as the score covers both the quality of the conducted study and the completeness of reporting. For example, if the context is not discussed because it seemed obvious, no points could be awarded.

Table C1: CHEC list assessment of the economic evaluations of OOH-PC

**Item**	**Checklist Question**	(Flaherty et al., 2022)	(Hansen & Munck, 1998)	(Moore et al., 2021)	(Broekman et al., 2017)	(Sterner et al., 2012)	(Lattimer et al., 2000)	(Patwardhan et al., 2012)	(Flymn, 1998)	(Poole et al., 1993)	(Reuter et al., 2016)	(Wijers et al., 2012)	(Moe et al., 2019)	(Chesteen et al., 1986)
1	Is the study population clearly described?	1	1	1	1	1	1	1	1	1	1	1	1	1
2	Are competing alternatives clearly described?	1	1	1	1	1	n/a	1	1	1	1	1	1	1
3	Is a well-defined research question posed in answerable form?	1	1	1	1	1	1	1	1	1	1	1	1	1
4	Is the economic study design appropriate to the stated objective?	n/a	n/a	n/a	n/a	n/a	n/a	n/a	n/a	n/a	n/a	n/a	n/a	n/a

Item	Checklist Question	(Flaherty et al., 2022)	(Hansen & Munck, 1998)	(Moore et al., 2021)	(Broekman et al., 2017)	(Sterner et al., 2012)	(Lattimer et al., 2000)	(Patwardhan et al., 2012)	(Flynn, 1998)	(Poole et al., 1993)	(Reuter et al., 2016)	(Wijers et al., 2012)	(Moe et al., 2019)	(Chesteen et al., 1986)
5	Is the chosen time horizon appropriate in order to include relevant costs and consequences?	1	1	1	1	1	1	1	1	1	1	1	1	1
6	Is the actual perspective chosen appropriate?	1	0	0	1	0	1	0	0	0	1	0	1	1
7	Are all important and relevant costs for each alternative identified?	1	0	1	1	1	1	1	1	1	1	0	1	1
8	Are all costs measured appropriately in physical units?	1	0	1	1	1	1	1	1	1	1	1	1	1
9	Are costs valued appropriately?	1	0	1	1	1	1	1	1	1	0	1	1	1
10	Are all important and relevant outcomes for each alternative identified?	1	1	1	1	1	n/a	1	1	1	1	1	1	1
11	Are all outcomes measured appropriately?	1	1	1	1	1	n/a	1	1	1	1	1	1	1
12	Are outcomes valued appropriately?*	1	1	1	1	1	n/a	1	1	1	1	1	1	1
13	Is an a appropriate incremental analysis of costs and outcomes of alternatives performed?	1	n/a	1	1	1	1	1	1	1	1	1	1	n/a
14	Are all future costs and outcomes discounted appropriately?	1	n/a	n/a	n/a	n/a	n/a	n/a	n/a	n/a	n/a	n/a	1	n/a
15	Are all important variables, whose values are uncertain, appropriately subjected to sensitivity analysis?	1	n/a	1	0	0	1	1	0	0	0	0	0	0
16	Do the conclusions follow from the data reported?	1	1	1	1	1	1	1	1	1	n/a	n/a	1	1
17	Does the study discuss the generalizability of the results to other settings and patient/client groups?	0	0	1	0	1	1	1	0	0	n/a	n/a	0	0
18	Does the article/report indicate that there is no potential conflict of interest (CoI) of study researcher(s) and funder(s)?	1	0	1	1	0	1	1	0	0	1	1	0	0
19	Are ethical and distributional issues discussed appropriately?	1	0	0	0	0	1	0	0	0	n/a	n/a	0	0
	Total score	16/ 18	8/ 15	15/17	14/ 17	13/ 17	13/13	15/17	12/17	12/17	12/14	11/14	14/18	12/16
	**Total score (%)**	**89%**	**53%**	**88%**	**82%**	**76%**	**100%**	**88%**	**71%**	**71%**	**86%**	**79%**	**77%**	**75%**

Appendix D: Details on the identified cost-effectiveness studies for OOH-PC

Table D1: Summary of 13 identified cost-effectiveness studies

**Year of publication**	**Country**	**Brief study overview**	**Comparators used**	**Effect measures**	**Reference**
2022	Haiti	An exploratory cost-effectiveness study compared a nighttime pre-emergency paediatric telemedicine together with a home delivery service to the hospital emergency	Hospital emergency department	Disability-adjusted life year (DALY) averted	(Flaherty et al., 2022)
2020	England and Wales	A Controlled observational study evaluated the cost-effectiveness of an intervention called AIMS, designed to treat alcohol intoxication during late nights, weekends, and holidays, compared to areas matched without AIMS	No intervention (i.e. No AIMS, which is an intervention designed to treat alcohol intoxication during out of hours)	This study estimated cost savings based on avoided ED visits. Others included the proportion of ED attendances achieving the 4-hour target for total time in the ED, the proportion of high priority ambulance calls achieving the 8-minute and 19-minute targets for ambulance response	(Moore et al., 2021)
2019	Canada	Assessed cost-savings (ROI) of After-Hours Clinics	No Intervention	Avoided ED visits	(Moe et al., 2019)
2017	The Netherlands	Using a cross-sectional observational design, this study evaluated the joint operation of OOH General Practitioners (GPs) and	A form of OOH-PC delivery where OOH GPs and the ED operate independently	Patient satisfaction and length of stay	(Broekman et al., 2017)

		Emergency Departments (EDs) with shared entrance and triage, comparing it to the usual care of independently operating OOH GPs and EDs			
2012	United States of America	A cross-sectional ROI study compared demographically and clinically matched nonemergent patients managed in an After-Hours Clinic versus a pediatric emergency department	Pediatric emergency department	Length of stay	(Sterner et al., 2012)
2012	United States of America	Assessed the Cost consequences of Convenient Care Clinics	"No Intervention"	Avoided ED visits	(Patwardhan et al., 2012)
2011	The Netherlands	A protocol that proposes an RCT to compare OOH-PC care provided by Nurse Practitioners (NPs) against OOH-PC care provided by General Practitioners.	A form of OOH-PC delivery where care is provided by General Practitioners.	Feasibility, type of consultation, duration of the consultation, type of care provider, number of patients, number of prescriptions, number of test & investigations, referral to other healthcare providers and the emergency department, Provider workload, Knowledge/competence of the NPs, Health Status (EQ-5D)	(Wijers et al., 2012)
2000	England	Through an ROI, assessed the costs and effects of nurse-led OOH telephone	A from OOH-PC delivery representing the usual	Net savings across the two interventions using emergency hospital admissions, hospital admission length of	(Lattimer et al., 2000)

		consultation using decision support software versus the usual GP-led OOH telephone consultation.	care provided by a general practice cooperative	stay, home visits by physicians, and attendance at the practice in three days	
1998	Denmark	Using a before-and-after approach, compared OOH-PC before and after structural reforms	A form of OOH-PC before the introduction of structural reforms	the number and type of patient contacts and patient satisfaction	(Hansen & Munck, 1998)
1998	The United States of America	Through a retrospective review of an after-hour telephone triage programme, assessed the costs averted through hospital use avoidance	No intervention	Net savings from avoided hospital utilization	(Flynn, 1998)
1996	France	A protocol that proposes an RCT to compare two modes of handling telephone calls in OOH. Telephone protocol driven telephone consultation versus standard of care telephone handling in the OOH	A form of handling OOH-PC telephone call representing the standard of care	Hospital admissions, Emergency department visits and mortality	(Reuter et al., 2016)
1993	The United States of America	Through a retrospective review of after-hour programme data, assessed the costs and effects of the application of an area-wide telephone triage and advice	No Intervention	Alongside program costs, presented, subscribing physician satisfaction, parent satisfaction, the accuracy and appropriateness of telephone triage.	(Poole et al., 1993)

		system for pediatric practices			
1986	The united Sates of America	Compared family practice centers providing extended hours to free-standing emergency care centers as sources of primary care services. .	Emergency department	Reported average costs and patient/provider satisfaction	(Chesteen et al., 1986)

References

Broekman S, Van Gils-Van Rooij E, Meijboom, B, De Bakker D and Yzermans C (2017) Do out-of-hours general practitioner services and emergency departments cost more by collaborating or by working separately? A cost analysis. Journal of Primary Health Care, 9, 212–219. https://doi.org/10.1071/HC17015

Chesteen SA, Warren SE., & Woolley FR (1986). A comparison of family practice clinics and free-standing emergency centers: organizational characteristics, process of care, and patient satisfaction. The Journal of Family Practice, 23, 377–382.

Drummond M, Sculpher M, Claxton K, Stoddart G and Torrance G (2015) Methods for the Economic Evaluation of Health Care Programmes (4th ed.). Oxford University Press, Oxford. https://books.google.co.uk/books?id=lvWACgAAQBAJ

Evers S, Goossens M, de Vet H, van Tulder M and Ament, A (2005) Criteria list for assessment of methodological quality of economic evaluations:consensus on health economic criteria. International Journal of Technology Assessment in Health Care, 21, 240–245. http://www.beoz.unimaas.nl/chec/.

Flaherty KE, Klarman MB, Cajusma Y, Schon J, Exantus L, Beau de Rochars VM, Baril C, Becker TK and Nelson EJ (2022) A Nighttime Telemedicine and Medication Delivery Service to Avert Pediatric Emergencies in Haiti: An Exploratory Cost-Effectiveness Analysis. The American Journal of Tropical Medicine and Hygiene, 106, 1063. https://doi.org/10.4269/AJTMH.21-1068

Flynn DM (1998) Telephone triage as a strategy to ensure 24-hour access to medical care after the closure of supporting medical activity Military Medicine, 163, 702–706.

Hansen BL and Munck A (1998) Out-of-hours service in Denmark: the effect of a structural change. The British Journal of General Practice 48, 1497-1499. /pmc/articles/PMC1313198/?report=abstract

Lattimer V, Sassi F, George S, Moore M, Turnbull J, Mullee M and Smith H (2000) Cost analysis of nurse telephone consultation in out of hours primary care: evidence from a randomised controlled trial. BMJ (Clinical Research Ed.), 320, 1053–1057. https://doi.org/10.1136/bmj.320.7241.1053

Moe J, Oland R and Moe G (2019) Impact of a Primary Care After-Hours Clinic on Avoidable Emergency Department Visits and Costs. Healthcare Quarterly, 22, 42–47. https://doi.org/10.12927/HCQ.2019.25837

Moore S, Young T, Irving A, Goodacre S, Brennan A and Amos Y (2021) Controlled observational study and economic evaluation of the effect of city-centre night-time alcohol intoxication management services on the emergency care system compared with usual care. Emergency Medicine Journal, 38, 504–510. https://doi.org/10.1136/EMERMED-2019-209273

Patwardhan A, Davis J, Murphy P and Ryan SF (2012) After-hours access of convenient care clinics and cost savings associated with avoidance of higher-cost sites of care. Http://Dx.Doi.Org/10.1177/2150131911436251, 3, 243–245. https://doi.org/10.1177/2150131911436251

Poole SR, Schmitt BD, Carruth T, Peterson-Smith A and Slusarski M (1993). After-hours telephone coverage: the application of an area-wide telephone triage and advice system for pediatric practices. Pediatrics, 92, 670–679. https://doi.org/10.1542/peds.92.5.670

Reuter P-G, Desmettre T, Guinemer S, Ducros O, Begey S, Ricard-Hibon A, Billier L, Grignon O, Megy-Michoux I, Latouff J-N., Sourbes A, Latier J, Durand-Zaleski I, Lapostolle F, Vicaut E and Adnet F (2016) Effectiveness and cost-effectiveness of telephone consultations for fever or gastroenteritis using a formalised procedure in general practice: study protocol of a cluster randomised controlled trial. Trials, 17, 461. https://doi.org/10.1186/s13063-016-1585-9

Søvsø MB, Christensen MB, Bech BH, Christensen HC, Christensen EF and Huibers L (2019) Contacting out-of-hours primary care or emergency medical services for time-critical conditions - impact on patient outcomes. BMC Health Services Research, 19, 1-10. https://doi.org/10.1186/S12913-019-4674-0

Sterner SE, Coco T, Monroe KW, King WD and Losek, JD (2012). A new after-hours clinic model provides cost-saving, faster care compared with a pediatric emergency department. Pediatric Emergency Care, 28, 1162–1165. https://doi.org/10.1097/PEC.0b013e318271733e

van Delft LCJ, Kelleners-Smeets NWJ, Peeters A, Mosterd K and Essers BAB (2023) A systematic review of economic evaluations for the interventions of superficial basal cell carcinoma. EJC Skin Cancer, 1, 100008. https://doi.org/10.1016/J.EJCSKN.2023.100008

van Mastrigt GAPG, Hiligsmann M, Arts JJC, Broos PH, Kleijnen J, Evers SMAA and Majoie MHJM (2016) How to prepare a systematic review of economic evaluations for informing evidence-based healthcare decisions: a five-step approach (part 1/3). Expert Review of Pharmacoeconomics & Outcomes Research, 16, 689–704. https://doi.org/10.1080/14737167.2016.1246960

Wijers N, Schoonhoven L, Giesen P, Vrijhoef H, Van Der Burgt R, Mintjes J, Wensing M and Laurant M (2012) The effectiveness of nurse practitioners working at a GP cooperative: a study protocol. BMC Family Practice, 13. https://doi.org/10.1186/1471-2296-13-75
